# Effects of *Fructus mume* Extract on MAPK and NF-****κ****B Signaling and the Resultant Improvement in the Cognitive Deficits Induced by Chronic Cerebral Hypoperfusion

**DOI:** 10.1155/2012/450838

**Published:** 2012-12-30

**Authors:** Won Kyung Jeon, Jinhua Ma, Bo-Ryoung Choi, Seol-Heui Han, Qinghao Jin, Bang Yeon Hwang, Jung-Soo Han

**Affiliations:** ^1^Herbal Medicine Research Division, Korea Institute of Oriental Medicine, Daejeon 305-811, Republic of Korea; ^2^Department of Biological Sciences, Konkuk University, 1 Hwayang-dong, Gwangjin-gu, Seoul 143-701, Republic of Korea; ^3^Department of Neurology, Konkuk University Hospital, Center for Geriatric Neuroscience Research, Institute of Biomedical Science and Technology, Konkuk University, Seoul 143-729, Republic of Korea; ^4^College of Pharmacy, Chungbuk National University, Cheongju 361-763, Republic of Korea

## Abstract

*Fructus mume* (*F. mume*) has been used as a medicinal food in Japan and has been reported to have anti-inflammatory effects in inflammatory bowel disease and macrophage-mediated inflammation. We investigated the effects of *F. mume* extracts on cognitive dysfunction in rats with chronic cerebral hypoperfusion and the molecular mechanisms underlying these effects. Chronic cerebral hypoperfusion was induced in male Wister rats by bilateral common artery occlusion (BCCAo). Daily administration of *F. mume* extracts was started on day 20 after post-BCCAo and continued for 40 days. The status of hippocampus-dependent memory was evaluated in control rats, rats with chronic cerebral hypoperfusion, and rats with chronic cerebral hypoperfusion that were administered *F. mume*. The levels of microglial activation were measured in the hippocampus and the fimbria of hippocampus, and expression levels of hippocampal mitogen-activated protein kinase (MAPK) and nuclear factor-**κ**B (NF-**κ**B) were examined. Rats that received chronic cerebral hypoperfusion showed spatial memory impairments relative to the control rats; these impairments were reduced by daily administration of *F. mume*. Administration of *F. mume* mitigated the microglial activation and alterations of hippocampal MAPK and NF-**κ**B signaling in the rats with chronic cerebral hypoperfusion. These results indicate that *F. mume* may possess therapeutic potential for the prevention of vascular dementia via inhibition of inflammatory processes.

## 1. Introduction

Disorders of cerebral circulation lead to the development of numerous neurological diseases [1]. A moderate but persistent reduction in cerebral blood flow contributes to prolonged cognitive decline and eventually results in the development and progression of vascular dementia (VaD) [2]. VaD is the second most common type of dementia following Alzheimer's disease (AD), and a pathological characteristic of brain in VaD is neuroinflammation, as seen in the brains of AD patients [3, 4]. Brain tissues from patients with VaD have activated microglia in areas with white matter damage [4–7].

Chronic cerebral hypoperfusion leads to the development of VaD [1]. Permanent bilateral common carotid artery occlusion (BCCAo) has been used extensively to study the role of chronic cerebral hypoperfusion in the development of cognitive decline in VaD, to reveal its underlying mechanism, and to further develop its therapeutic treatments [8–10]. Chronic BCCAo reduces cerebral blood flow and results in cognitive decline, glial activation, and white matter damage similar to those occurring in VaD [11–13]. 

MAPK signaling plays a crucial role in signal transduction and in the development of AD [14]. A recent study reported that MAPK signaling was altered in the hippocampi of rats that showed spatial memory impairments induced by chronic BCCAo [15, 16]. In particular, the expression levels of phosphorylated extracellular signal-regulated kinase (p-ERK), a subfamily number of MAPK, were increased in the hippocampi of rats that underwent chronic BCCAo. NF-*κ*B activity is altered in association with the brain pathology seen in chronic neurodegenerative disorders [17]. NF-*κ*B has been long known to critically regulate apoptosis. In particular, p65, an NF-*κ*B DNA-binding subunit, has been reported to be associated with spatial memory [18]. In the brains of AD patients, levels of p65 immunoactivity are increased in neurons and astrocytes that are in close vicinity of early plaques [19]. Moreover, the brains of rats with chronic cerebral hypoperfusion show increased immunoreactivity of p65, primarily in astrocytes, and low expression in neurons [20].

*Fructus mume* (*F. mume*), the processed unripe fruit of *Prunus mume, *has been used in the treatment of chronic diarrhea and lingering dysentery in Asian countries. Recently, it was reported that *F. mume *extract is an effective treatment for colitis in an animal model [21]. Choi et al. (2007) also examined therapeutic mechanisms and effects of *F. mume* extract on macrophage-mediated inflammation by examining whether treatment with *F. mume* inhibited proinflammatory mediators in lipopolysaccharide- (LPS-) stimulated RAW 264.7 cells [22]. Treatment with *F. mume* inhibited the LPS-induced production of nitric oxide, prostaglandin E2, and interleukin-6 productions. *F. mume* was found to suppress activation of mitogen-activated protein kinase (MAPK) and nuclear factor-*κ*B (NF-*κ*B), both of which are induced by LPS stimulation. These studies indicate that *F. mume *is a good candidate to be an anti-inflammatory agent. 

Therefore, the present study was conducted to examine the effects of *F. mume* extract on spatial memory impairments and microglial activation induced by chronic BCCAo. Additionally, the status of p-ERK and NF-*κ*B p65 expression were evaluated in the hippocampi of BCCAo rats and BCCAo rats treated with *F. mume* extract. 

## 2. Material and Methods

### 2.1. Animals

Fifty-eight male Wistar rats were used in a chronic BCCAo experiment (12 weeks old; Charles River Co., Gapeung, Republic of Korea). The rats were housed in a vivarium at Konkuk University for two weeks at the beginning of the experiment under controlled temperature (22 ± 1°C) and humidity (50 ± 10%) on a 12-h light/dark cycle (lights on at 08:00 h). Food and water were provided ad libitum to all animals throughout the experiment. The Institutional Animal Care and Use Committee of Konkuk University approved all protocols described in the report. All surgical procedures and behavioral testing took place during the light phase.

### 2.2. Surgery

The Wistar rats were anesthetized using a mixture of 5% isoflurane and oxygen, and anesthesia was maintained with 3% isoflurane during the surgical procedure. A midline incision was made to expose both common carotid arteries, which were then tightly double ligated with silk sutures. In addition, control animals were subjected to a sham operation in which they underwent the same procedure without BCCAo. The rectal temperature was maintained at 37.0 ± 0.5°C with a heating pad throughout the surgical procedure. During hypoperfusion, about 5% of the animals showed neurological symptoms such as seizures with squatting, and these animals died within one week after postsurgery. In addition, rats that lost at least 80% of their presurgical weight during a drug or vehicle administration were excluded from further experiments.

### 2.3. Preparation of *F. mume* Extracts and Administration

*F. mume* was purchased from a commercial supplier (Kwangmyung-Dang, Ulsan, Korea) in 2010. It was identified by the Herbal Quality Control Team and deposited at the Creative Research Laboratory, KIOM (Korea). Dried *F. mume *was pulverized and extracted with distilled water (2 kg/8 L) for 2 h below 100°C in an ultrasound-assisted extractor (OM30-EP; Sonimedi, Korea). All extracts were concentrated under vacuum using a rotary evaporator after filtration and were then dried for 48 h at 40°C by using an extract vacuum drier (Exdryer, Sonimedi, Korea) to yield a powder extract (324.5 g, 16.225% yield). The powder extract was suspended in sterilized distilled water at the appropriate concentrations. An HPLC assay was performed with citric acid as a standard maker for quality control of the *F. mume *extract composition in each experiment. HPLC was performed using two Waters 515 pumps, a 2996 photodiode array detector, and a Phenomenex Synergi Hydro RP-80A (4 m, 4.6 × 250 mm i.d.). The mobile phase was composed of acetonitrile (A) and 0.1% phosphoric acid (B) with a linear gradient elution: 0 min, 100% B; 15 min, 3.8% A. *F. mume *extract was filtered on membrane filters with a 0.45 mm pore size (Millipore) and a 10 L injection volume. Citric acid was detected at a wavelength of 220 nm. The crude extract was analyzed in triplicate, and the citric acid content was found to be about 18.68%.

**Figure 1:**
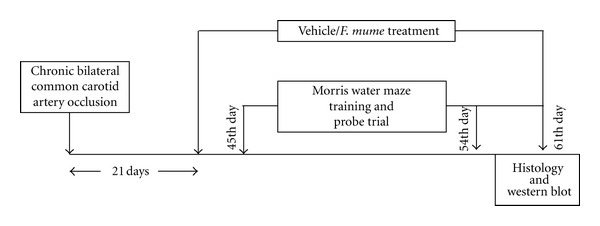
Experimental design.

The rats used in the BCCAo experiment were segregated into five groups: a sham-operated group (oral administration of the drug vehicle, *n* = 14); BCCAo group (oral administration of the drug vehicle, *n* = 14); three BCCAo groups that received daily oral administration of drugs (100 mg/kg* F. mume *extract, 200 mg/kg* F. mume *extract, and 400 mg/kg* F. mume *extract, *n* = 10 rats per group). Vehicle/drug treatment was started on 20th day after BCCAo surgery and was continued until the end of the experiment (see Figure 1). During drug administration, two rats were lost in each* F. mume *extract treatment group due to the stress related to long-term oral feeding, but the extract of *F. mume *showed no toxicity in terms of general behavioral change and mortality.

### 2.4. HPLC Analysis of Water Extract of the Fruits of *F. mume*

An HPLC was performed using a Waters alliance 2695 high performance liquid chromatography (HPLC) system, with a 2996 PDA detector and a YMC Hydrosphere C-18 (5 mm, 4.6 × 250 mm i.d.) (YMC Co. Ltd., Tokyo, Japan). The mobile phase was composed of acetonitrile (A) and water (B) with a step gradient elution: (A)/(B) = 0/100 (0 min), (A)/(B) = 10/90 (15 min), and (A)/(B) = 100/0 (40 min; hold for 10 min). The flow rate was 1.0 mL/min. The crude water extract of *F. mume* was filtered on membrane filters with a prose size 0.45 mm (Millipore), and the injection volume was 10 *μ*L.

### 2.5. Behavioral Assessment

The apparatus was as follow. The maze was a round tank, 1.83 m in diameter and 58 cm deep, and filled to a depth of 35.5 cm with tepid (26 ± 1°C) water made opaque by the addition of white paint (tempera). A moveable circular platform, 12 cm in diameter, was located 2 cm below the surface of the water. The maze was surrounded by white curtains, on which black cloths of various shapes and sizes were placed as visual stimuli. A camera located above the center of the maze relayed images to a videocassette recorder and an HVS Image Analysis Computer System. Data from the water maze trials were analyzed using software provided by HVS (Hampton, United Kingdom).

The training procedure of the spatial memory task for chronic BCCAo rats has been described previously [15]. In a standardized procedure that required the use of distal cues in a maze environment, the rats were trained to learn the position of a camouflaged escape platform [23]. Briefly, every session contained five trials across two days, and the total training was composed of four sessions. During each training trial, the location of the platform remained constant, and the rats swam for 90 s or until they found the platform. Across the trials, the starting location varied among four equidistant points around the perimeter of the apparatus. A probe trial was conducted 30 min after the second session and the fourth session to assess the development of spatial bias in the maze; thus, the entire training procedure included two probe trials for each rat. During these probe trials, the rats swam with the platform retracted to the bottom of the pool for 30 s, at which time the platform was raised to its normal position for completion of the trial. The behavioral assessment was started 45 days after surgery (see Figure 1).

### 2.6. Western Blot Analysis

Rats used in the western blot analysis were decapitated one week after the behavioral experiment, and the hippocampi of the brains were microdissected and then frozen. Whole tissue extracts were prepared using a protein extraction method described elsewhere [24]. For total protein extracts, individual tissue samples were weighed and then homogenized in five volumes of ice-cold buffer containing 20 mM Tris at pH 7.5, 5% glycerol, 1.5 mM EDTA, 40 mM KCl, 0.5 mM dithiothreitol, and protease inhibitors (no. 539131, Calbiochem). The homogenates were then centrifuged at 20,800 ×g  for 1 h at 4°C, and the supernatant was harvested, snap-frozen, and stored at −80°C. Cytosolic or nuclear protein extracts were prepared using a previously described protein extraction method [25]. Individual frozen tissues were weighed and homogenized (1 : 2 = tissue mass :  volume) in ice-cold 20 mM Tris-HCl (pH 7.5) buffer containing 10% glycerol, 50 mM NaCl, 1 mM EDTA, 1 mM EGTA, 2 mM dithiothreitol, protease inhibitor cocktail (no. 539131, Calbiochem), and phosphatase inhibitors (1 mM PMSF, 2 mM Na_3_VO_4_, and 25 mM NaF) with 20 strokes in a tissue grinder (no. 440613, Radnoti). Samples were centrifuged 10 min at 2,000 ×g at 4°C; supernatants were centrifuged for 1 h at 105,000 ×g, and final supernatants were used as the cytoplasmic fraction. Pellets were washed in 0.5 mL of homogenization buffer and centrifuged for 10 min at 2,000 ×g at 4°C. Final pellets were weighed, resuspended (1 : 1 = mass : volume) in the same buffer supplied with 0.5 M KCl, incubated for 1 h in an ice bath (with frequent vortexing), and centrifuged for 10 min at 8,000 ×g at 4°C. The supernatant was used as a nuclear extract. Protein concentrations of these extracts were determined by the Bradford assay. 

Protein standards (Precision Plus Protein Western C standards for SDS-PAGE; Bio-Rad) were loaded on the first lane of each gel (left side only). The proteins were then separated by SDS-PAGE and transferred to a PVDF membrane by electroblotting using the Mini Trans-Blot Cell (Bio-Rad). This membrane was incubated with a primary antibody (Ab) against ERK (1 : 5,000, Cell Signaling), p-ERK (1 : 3,000, Cell Signaling), actin (1 : 5,000), I*κ*B-*α* (1 : 1,000), and NF-*κ*B p65 (1 : 1,000, Upstate Biotechnology) and then with HRP-conjugated secondary Abs. The membranes were visualized using an ECL system and developed onto hyperfilm. All experiments were repeated three or more times with anti-actin antibody as the loading control. The relative expression levels of ERK, p-ERK, I*κ*B-*α*, and NF-*κ*B p65 were determined by densitometry and normalization to *β*-actin (1 : 5,000, Sigma), an invariant cytoskeletal protein. Each group for western blot analyses had 3–6 rats per group. 

### 2.7. Immunohistology

Rats were euthanized by lethal overdose of ketamine HCl (30 mg/kg) and xylazine (2.5 mg/kg) one week after the behavioral experiments, after which they were intracardially perfused with 4% paraformaldehyde in a 0.1 M phosphate buffer (pH 7.4, PPB). Following fixation, the brains were removed, postfixed in PPB (2 h), treated with PBS containing 20% sucrose for cryoprotection (24 h), frozen on powdered dry ice, and then sectioned (coronal plane: 40 um) using a microtome. Eighth section was used for immunohistochemical analysis of OX-6 and ionized calcium-binding adaptor molecule (Iba-1). Iba-1 is a 17-kDa EF-hand protein whose expression is restricted to microglia/macrophages [26]. In immunostaining of OX-6 or Iba-1, endogenous peroxidase activity in free-floating sections was quenched by 30 min of incubation in 3% H_2_O_2_/10% MeOH in PBS. The sections were then incubated for 1 h at room temperature in PBS with 0.3% Triton-X 100 containing 10% fetal horse serum (GIBCO). The sections were then incubated with OX-6 (mouse anti-OX-6, BD Bioscience, 1 : 1000) or Iba-1 antibody (rabbit anti-Iba-1 polyclonal antibody, Wako, 1 : 1,000) for 1 h at room temperature and overnight at 4°C in 3% serum in PBS-T solution. The sections were then incubated for 1 h with the appropriate biotinylated secondary antibodies (Vector,  1 : 200) and for 1 h in ExtrAvidin peroxidase conjugate (Sigma Aldrich, 1 : 1,000). Finally, the sections were reacted with a Vector SG substrate kit (Sigma Aldrich) for peroxidase and mounted onto resin coated slides, after which they were dried for up to one week. Dried sections on slides were coverslipped with Permount reagent. 

To evaluate colocalization of p-ERK with neuron and translocation of NF-*κ*B into nucleus, immunofluorescence study was performed. Sections were washed in PBS with 0.3% Triton X-100 and then incubated in blocking serum, 5% normal horse serum in 0.15% triton with PBS. Subsequently, sections were incubated in primary antibody solution for 20 hours at room temperature. For primary antibody, mouse anti-NeuN antibody (Millipore, 1 : 1000), rabbit Phospho-p44/42 MAPK (Erk1/2) antibody (Cell Signaling, 1 : 100), and rabbit anti NF-*κ*B antibody (Santacruz, 1 : 500) were used. Sections were washed in PBS with 0.15% Triton X-100 and incubated in a secondary antibody solution (Alexa 568 conjugated donkey anti-rabbit antibody, Alexa 488 conjugated donkey anti-mouse antibody, 1 : 200) for 2 hours at room temperature. Stained sections were mounted on resin-coated slides and dried for 30 min. Slides were then cover slipped with ProLong Gold antifade reagent (Invitrogen).

To quantify microglial cells, the number of OX-6 or Iba-1 positive cells was counted. Sections including hippocampus and the fimbria of the hippocampus from 3–6 rats per group were analyzed. For a quantitative analysis, we selected specific regions in which neuroinflammatory changes had been reported in the literature [27]. The hippocampus, including CA1, CA3, and dentate gyrus (DG), was the focus for quantification of Iba-1 positive cells. One region of interest (ROI) of 0.03 mm^2^ per one section in the hippocampal subregion, CA1, CA3, and DG (bregma −3.00 to −4.00 mm; six sections per rat) were selected. The number of Iba-1 microglial cells was counted in each ROI and averaged. ROI in the fimbria of the hippocampus (bregma −3.36 to −3.72 mm; 6 sections per rat) was selected. The number of OX-6 microglial cells was counted in each ROI and averaged. Hippocampal CA1, CA3, and the dentate gyrus were the focus for the quantification of p-ERK expression. Neuron layers in the CA1, CA3, and DG of the hippocampus (bregma −3.24 to −3.72 mm; 3 sections per rat) were selected. The integrity of p-ERK expression signal in each ROI was measured and averaged. 

### 2.8. Statistical Analysis

One-way ANOVA and one-way repeated ANOVA were conducted to assess the effects of *F. mume *extract on the changes in the expression levels of ERK, p-ERK, I*κ*B-*α*, cytosolic NF-*κ*B p65, nuclear NF-*κ*B p65, the number of OX-6 and Iba-1 positive cells, and the impairment of spatial memory induced by chronic BCCAo. Post hoc analyses (Dunnett *t* or *t*-test) were subsequently conducted to determine the effects of the *F. mume* treatment. *P* values of less than 0.05 were considered significant, unless otherwise specified. *t*-test was conducted to assess the effect of chronic BCCAo on intensity levels of p-ERK positive cells in hippocampus.

## 3. Results

### 3.1. HPLC Analysis of Water Extract of the Fruits of *F. mume*

**Figure 2:**
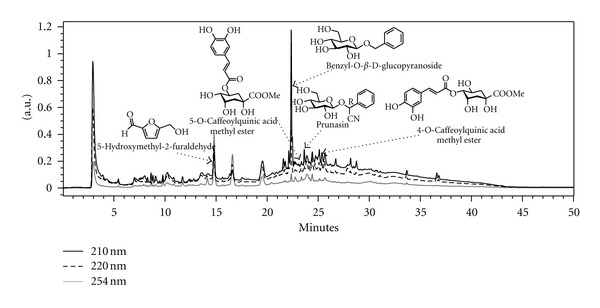
HPLC chromatogram of water extract of the fruits of *F. mume*.

As shown in Figure 2, the water extract of *F. mume* contained 5-hydroxymethyl-2-furaldehyde (**1**), 4-O-caffeoylquinic acid methyl ester (**2**), prunasin (**3**), 5-O-caffeoylquinic acid methyl ester (**4**), and benzyl-O-*β*-D-glucopyranoside (**5**), which were previously isolated from the water extract of the fruit of *F. mume* [28].

### 3.2. *F. mume* Extract Ameliorated Chronic BCCAo-Induced Spatial Memory Impairments

**Figure 3:**
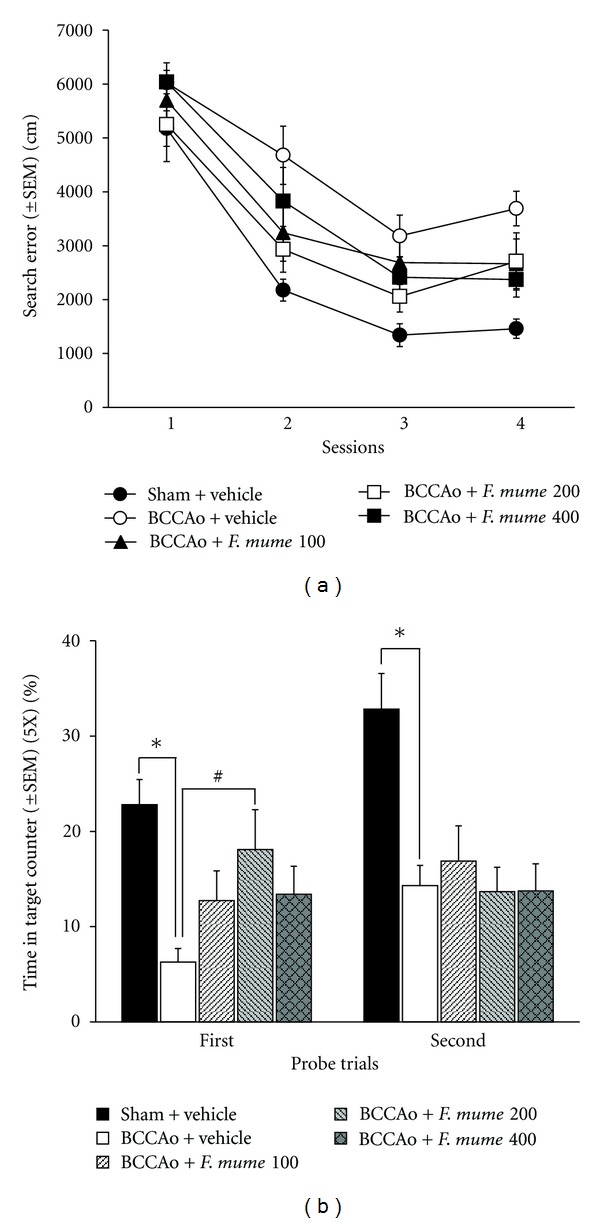
*F. mume *improves spatial memory impairment induced by chronic BCCAo. (a) Search error measure of spatial learning during five trial sessions of training trials. Compared to the learning in sham-operated control rats (sham + vehicle), learning was significantly impaired in the BCCAo rats (BCCAo + Vehicle). *F. mume* (BCCAo +* F. mume* 200 mg/kg) treatment significantly ameliorated the learning impairment induced by chronic BCCAo. (b) The percentage of time spent in the target annulus (5× of the platform size). In both probe trials, sham-operated control rats showed spatial bias, whereas the BCCAo rats did not (*). Compared to the BCCAo rats, *F. mume *treatment (BCCAo +* F. mume* 200) significantly showed spatial bias in the first probe trial (#).

In the Morris water maze task, search errors that are described in detail elsewhere [23] were used to assess the performance accuracy of spatial learning in the water maze. During each trial, the distance of the rats from the escape platform was sampled 10 times per sec, and these values were averaged in 1-sec bins. The cumulative search error was then calculated as the summed 1-sec averages of the proximity measures corrected for the particular start location on each trial. One-way repeated ANOVA showed that the effects of between groups (sham-operated control, BCCAo with vehicle, and BCCAo with drug treatments) were significant (*F*_(4,46)_ = 8.81, *P* < 0.001), as were the training effects (sessions) (*F*_(3,138)_ = 88.81, *P* < 0.001). The interaction effects between the group and training session were not significant. As shown in Figure 3(a), the sham-operated control rats quickly became proficient at locating the submerged platform during the training sessions; however, in comparison, the BCCAo rats did not show much improvement over the course of training. This was confirmed by post hoc analysis, which revealed significant differences between the sham-operated control rats and the BCCAo rats (*P* < 0.01). In addition, the BCCAo rats that were administered *F. mume *extract (200 mg/kg) showed significantly better performances than the BCCAo rats (*P* < 0.05). However, the BCCAo rats treated with *F. mume *extract (100 mg/kg or 400 mg/kg) showed no significant improvements in performance when compared with the BCCAo rats.

In addition, apparent differences in performance during the first probe trial were observed, as assessed by the percentage of time spent in the target annulus (5× of the platform size) during the 30-sec probe. The one-way ANOVAs for the first probe trial showed that the effects of between groups were significant (*F*_(4,46)_ = 6.92, *P* < 0.001). Post hoc analysis revealed that the sham-operated control rats had the spatial bias when compared with the vehicle-treated BCCAo rats (*P* < 0.01) (Figure 3(b)). Similar to the results of training, the BCCAo rats that were treated with *F. mume *extract (200 mg/kg) performed significantly better than the vehicle-treated BCCAo rats (*P* < 0.05). However, the BCCAo rats treated with *F. mume *extract (100 mg/kg or 400 mg/kg) showed no significant improvements in performance when compared to the vehicle-treated BCCAo rats. The one-way ANOVAs for the second probe trial showed that the effects of between groups were significant (*F*_(4,46)_ = 8.14, *P* < 0.001). Post hoc analysis revealed that the sham-operated control rats had the spatial bias when compared with the vehicle-treated BCCAo rats (*P* < 0.05) (Figure 3(b)). However, BCCAo rats subjected to drug treatments did not show statistically significant ameliorative effects during the second probe trial when compared with BCCAo rats that were treated with vehicle.

### 3.3. *F. mume* Extract Reduced BCCAo-Induced Microglial Activation in the Hippocampus

**Figure 4:**
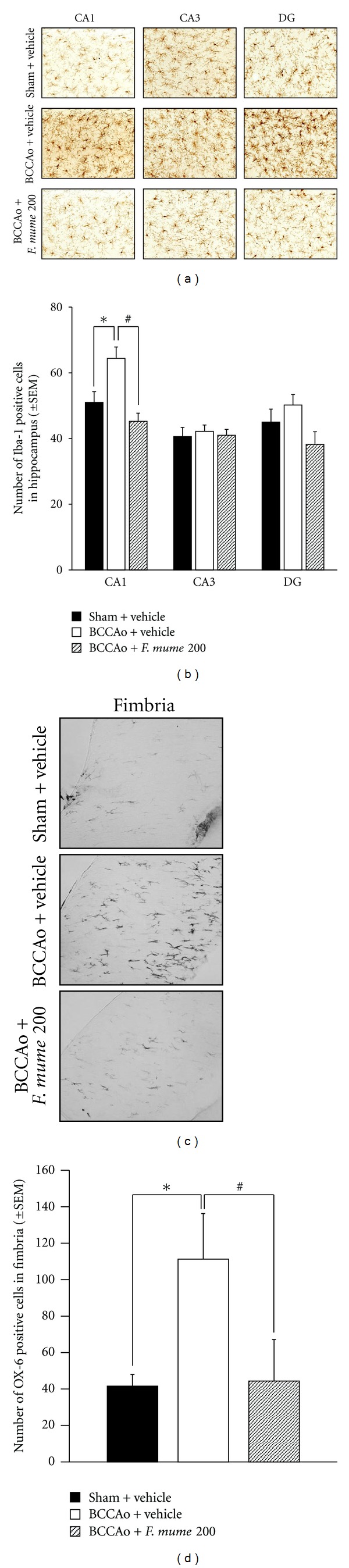
*F. mume* reduced BCCAo-induced microglial activation in the hippocampus and the fimbria of hippocampus. (a) Representative photomicrograph of Iba-1 positive cells. (b) The number of Iba-1 positive cells was increased in the CA1 of BCCAo rats (*), but not in the BCCAo hippocampus treated with *F. mume *(#). No difference among groups was observed in the CA3 and DG. (c) Representative photomicrograph of OX-6 positive cells microglia in the fimbria of the hippocampus. (d) The number of OX-6 positive cells was increased in the fimbria of BCCAo rats (*), but not in the BCCAo fimbria treated with *F. mume *(#).

Expression of Iba-1 is upregulated in activated microglia in response to brain disease [29]. To quantify the effects of *F. mume* treatment, the number of Iba-1 positive microglia in the subregion of the hippocampus (CA1, CA3, and DG) from every rat were counted in drawings of identical sections. ANOVA revealed significant group effects in the CA1 (*F*_(2,11)_ = 9.29, *P* < 0.05). Post hoc analyses of the group effects revealed that the number of Iba-1 positive cells in the BCCAo hippocampus was significantly higher than that in the control. The number of Iba-1 positive cells was less in the BCCAo hippocampus treated with *F. mume* than those in the vehicle-treated BCCAo hippocampus (*P* < 0.05) (Figures 4(a) and 4(b)). ANOVA analysis of CA3 and DG showed no group effects. 

In addition, it is reported that, upon staining for MHC class II antigen (OX-6), the number of OX-6 positive cells was increased in the fimbria of the hippocampus in rats with chronic BCCAo [16, 30]. The result of present experiment was consistent with those of the earlier reports. ANOVA revealed significant group effects in the fimbria (*F*_(2,12)_ = 4.42, *P* < 0.05). Post hoc analyses of the group effects revealed that the number of OX-6 positive cells in the fimbria with BCCAo was significantly higher than that in the control. The number of OX-6 positive cells was less in the BCCAo fimbria treated with *F. mume* than those in the vehicle-treated BCCAo hippocampus (*P* < 0.05) (Figures 4(c) and 4(d)). 

### 3.4. *F. mume* Extract Prevented BCCAo-Induced Increases of p-ERK Expression in the Hippocampus

**Figure 5:**
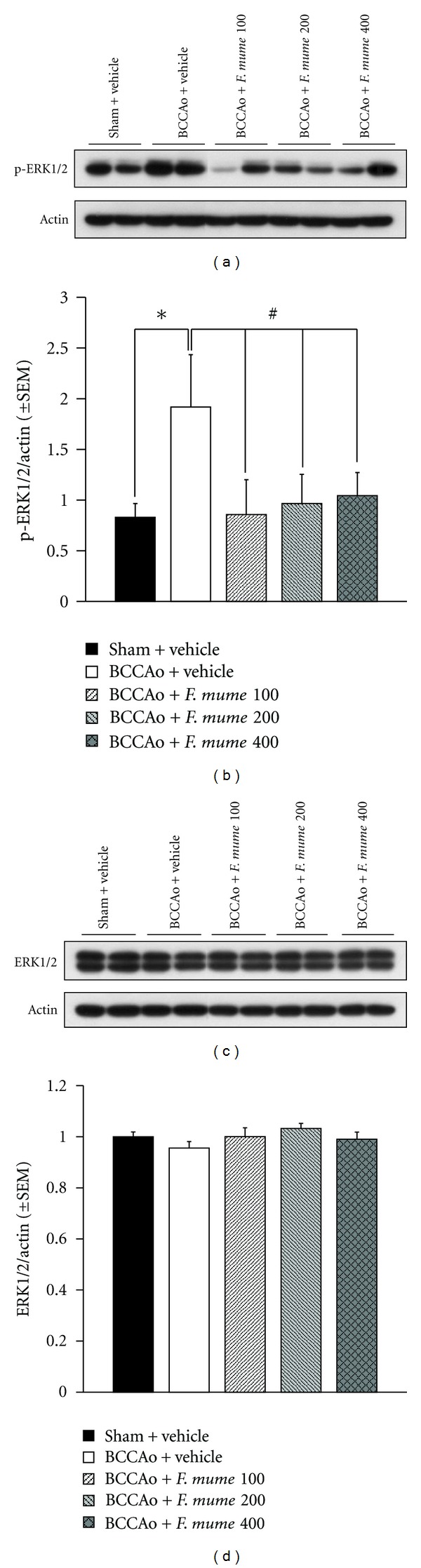
Treatment with *F. mume* prevented increases of p-ERK in the chronic BCCAo hippocampus. (a) Representative western blot of p-ERK (top) and actin (bottom). (b) Levels of p-ERK were increased in the hippocampi with chronic BCCAo relative to the control (*), and p-ERK levels were not increased in the hippocampi treated with *F. mume*, compared to those in the BCCAo hippocampus (#). (c) Representative western blot of ERK (top) and actin (bottom) (*). (d) No differences in ERK expression were detected among the groups.

MAPK signaling plays a major role in synaptic plasticity and hippocampus-dependent memory [31]. A recent study demonstrated that the levels of p-ERK were increased in the BCCAo hippocampus [15, 16]. Therefore, we decided to determine if *F. mume *extract reduced BCCAo-induced increases of p-ERK in the hippocampus. Specifically, western blot was used to measure the hippocampal p-ERK levels in the brains of rats in five groups, namely, sham-operated control, BCCAo + vehicle, BCCAo + *F. mume *extract (100 mg/kg), BCCAo + *F. mume *extract (200 mg/kg), and BCCAo + *F. mume *extract (400 mg/kg). One-way ANOVA of the p-ERK levels showed that the effects of between groups were significant (*F*_(4,11)_ = 3.15, *P* < 0.05). According to the post hoc analysis, the hippocampal p-ERK levels in the BCCAo rats were strongly upregulated when compared with those in the sham-operated control (Figures 5(a) and 5(b); *P* < 0.05). However, these BCCAo-induced increases were reduced by treatment with *F. mume *(*P* < 0.05). One-way ANOVA of ERK levels showed no group effects (Figures 5(c) and 5(d)). 

**Figure 6:**
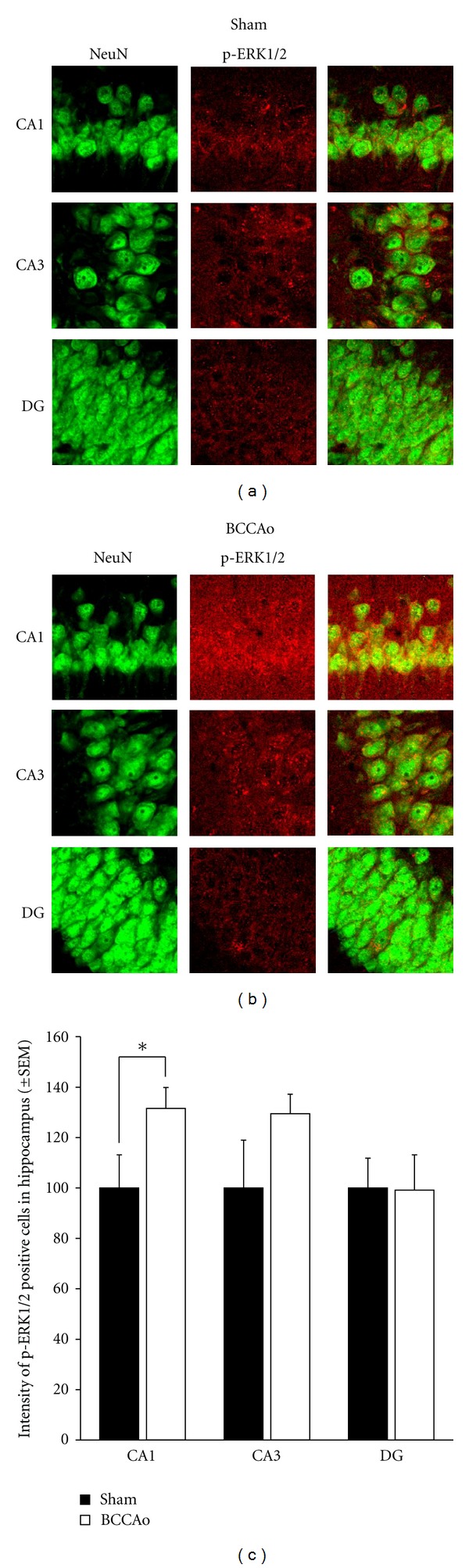
Expression of p-ERK in hippocampus cell layers (CA1, CA3, and DG) in the sham (a) and BCCAo (b). Signals of p-ERK were colocalized with neuron, and intensity of p-ERK positive signal in the hippocampus was significantly increased in CA1, not in CA3 and DG, layer of BCCAo group, compared to sham (c) (×200).

The subsequent experiment was conducted to examine if increases of p-ERK levels induced by chronic BCCAo occurred in the hippocampal CA1 neurons, using double immunofluorescence histochemistry. Figures 6(a) and 6(b) showed that the p-ERK positive signals were located in pyramidal cells in the hippocampal CA1 and CA3 and in the hilar region of the DG areas in sham-operated control and BCCAo rats. The effects of chronic BCCAo on p-ERK expression in the hippocampal areas were quantified by averaging the intensity of p-ERK positive cells (Figures 6(a) and 6(b)). Double immunofluorescence histochemistry showed that signals of p-ERK were more intense in NeuN positive cells of the hippocampal CA1 of rats with BCCAo than sham-operated control (Figure 6(c)). *t*-test of hippocampal CA1 p-ERK levels showed significant difference between groups (*P* < 0.05). *t*-test of hippocampal CA3 and DG p-ERK levels showed no differences between groups.

### 3.5. *F. mume* Extract Prevented BCCAo-Induced NF-*κ*B Activation

**Figure 7:**
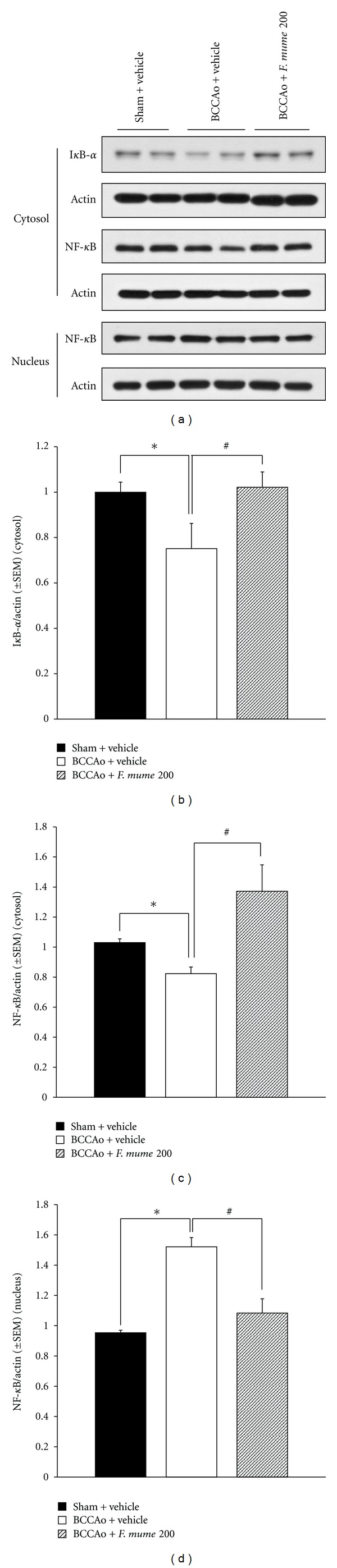
*F. mume *prevented BCCAo-induced NF-*κ*B activation. (a) Representative western blot of cytosolic I*κ*B-*α* (top) cytosolic NF-*κ*B p65 (middle), and nuclear NF-*κ*B p65 (bottom). (b, c) Cytosolic I*κ*B-*α* and cytosolic NF-*κ*B p65 were significantly diminished in the BCCAo hippocampus (*). *F. mume* prevented the attenuation of cytosolic I*κ*B-*α* and cytosolic NF-*κ*B p65 (#) (d). Levels of nuclear NF-*κ*B p65 were increased in the BCCAo hippocampus (*). Levels of nuclear NF-*κ*B p65 were not increased in the BCCAo hippocampus treated with *F. mume* (#).

NF*-κ*B signaling is altered in chronic neurodegenerative disorders, including AD [17]. Specifically, levels of NF*-κ*B p65 immunoactivity are increased in the brains of patients with AD [19]. Thus, we decided to determine if NF*-κ*B activation occurred in the hippocampus of rats with BCCAo and if NF*-κ*B activation was attenuated in the hippocampus of BCCAo rats treated with *F. mume *extract (200 mg/kg). One-way ANOVA of the levels of cytosolic NF-*κ*B p65, nuclear NF-*κ*B p65, and cytosolic I*κ*B-*α* of the hippocampus showed that the effects of between groups were significant (*F*_(2,9)_ ≥ 3.81, *P* ≤ 0.05). As assessed by western blotting and further analyzed in post hoc analyses, in cytosolic hippocampal extracts, BCCAo led to a decrease in the levels of the p65 subunit of the NF-*κ*B band (65 kDa) in the hippocampus (*P* < 0.05) (Figures 7(a) and 7(b)), as well as in the I*κ*B-*α* levels (41 kDa) (*P* < 0.05) (Figure 7(c)), while the NF-*κ*B p65 levels of nuclear hippocampal extracts were higher than those in the sham-operated control (*P* < 0.05) (Figure 7(d)), indicating that BCCAo increased NF-*κ*B translocation. *F. mume *extract (200 mg/kg) treatment reduced the translocation of NF-*κ*B p65 in the BCCAo hippocampus. 

**Figure 8:**
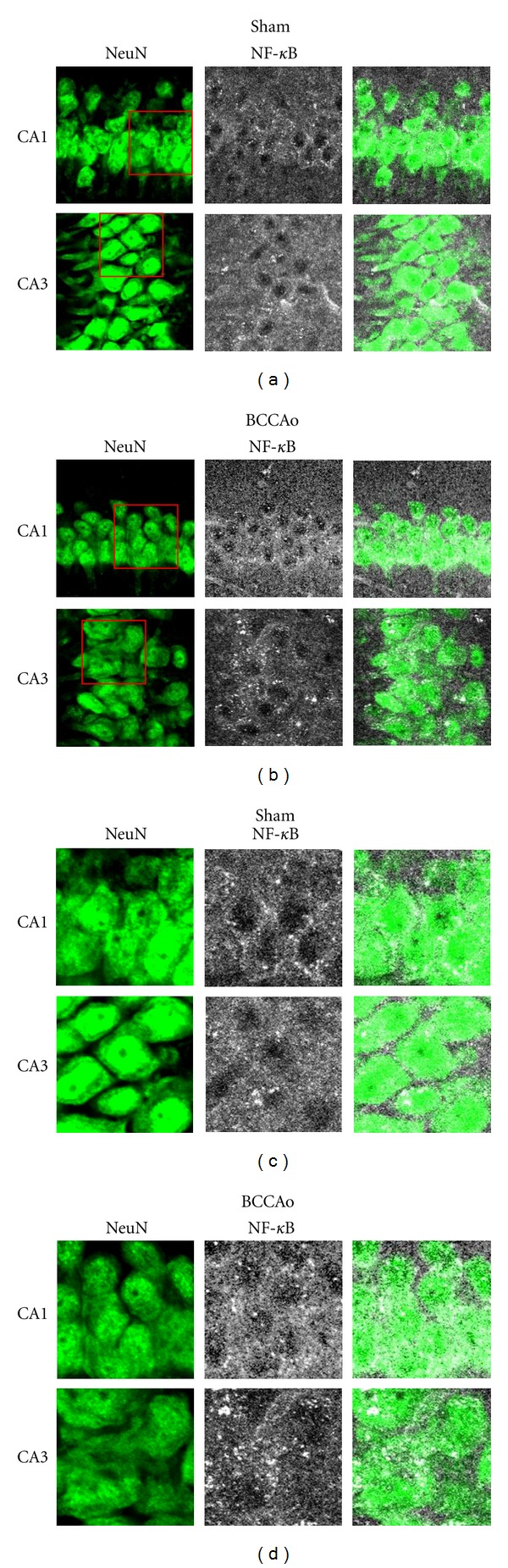
Low magnification (×200, a and b) and high magnification (×400, c and d) of NeuN (left) and NF-*κ*B positive signal (middle) in the hippocampus CA1 and CA3 cell layers in the sham (a and c) and BCCAo (b and d) groups. In the BCCAo group, signals of NF-*κ*B were translocated into nucleus, while most NF-*κ*B positive signals in sham group were located in cytosol.

The subsequent experiment was conducted to examine if NF-*κ*B translocation induced by chronic BCCAo occurred in the hippocampal CA1 and CA3 neurons, using double immunofluorescence histochemistry.  Figure 8 showed that the NF-*κ*B p65 positive signals were located in pyramidal cells in the hippocampal CA1 and CA3 in sham-operated control and BCCAo rats. Double immunofluorescence histochemistry showed that the NF-*κ*B p65 positive signals were more intense in NeuN positive cells of the hippocampal CA1 and CA3 of rats with BCCAo than sham-operated control (Figure 8(d)), suggesting that the NF-*κ*B had translocation to the nucleus in hippocampal CA1 and CA3 of brains with chronic BCCAo.

## 4. Discussion

*F. mume*, the smoked fruit of *Prunus mume *SIEB. *et* Zucc. (Rosaceae family), has been used for thousands of years in Asian countries to relieve cough, treat ulcers, and improve digestive function [21]. According to results of HPLC chromatogram, *F. mume *contains 5-hydroxymethyl-2-furaldehyde, 4-O-caffeoylquinic acid methyl ester, prunasin, 5-O-caffeoylquinic acid methyl ester, and benzyl-O-*β*-D-glucopyranoside, which is consistent with the earlier reports. Recent studies have shown that *F. mume* has antibacterial effects, anti-inflammatory effects, and therapeutic effects for gastrointestinal diseases [21, 22, 32] with its extract or single components. 

For example, 5-O-caffeoylquinic acid, the constituent of the fruit of *Prunus mume, *inhibited prostaglandin E_2_ production in the abdominal cavities of mice [33], indicating that there is a relationship between 5-O-caffeoylquinic acid and anti-inflammatory activities [34]. In addition, it is reported that 4-O-caffeoylquinic acid and 5-O-caffeoylquinic acid methyl ester, constituents of *F. mume,* showed inhibitory activity on advanced glycation end products (AGEs) [35]. The formation of AGEs is accelerated in age-related disease, and the receptor of AGEs has been increased in AD and VaD [36]. However, to date, using animal model, no study has examined the effectiveness of *F. mume* for neurological diseases, including AD and VaD. 

Thus, the present study is the first study to demonstrate the effectiveness of the *F. mume* extract in treating neurological diseases such as VaD. Specifically, *F. mume* extract ameliorated cognitive deficits induced by chronic cerebral hypoperfusion in rats. In addition, in order to reveal the molecular mechanisms of its action, the present study investigated ERK phosphorylation and NF-*κ*B signaling pathway. As reported previously, chronic BCCAo increased expression of p-ERK in the hippocampus, which was lessened by the treatment with *F. mume* [15]. Similar to its effect on hippocampal ERK signaling, BCCAo increased the activity of NF-*κ*B in the hippocampus, which is consistent with the finding of an earlier report [20]. The increased NF-*κ*B activity was reduced by treatment with *F. mume.*

The study demonstrating the anti-inflammatory effects of *F. mume* extract on LPS-stimulated RAW 264.7 cells also investigated its therapeutic mechanisms. *F. mume* inhibited LPS-induced phosphorylation of ERK and NF-*κ*B activation [22]. Another recent study reported that MAPK signaling was altered in the hippocampus of rats with chronic BCCAo [15]. Compared with the control rats, rats with chronic BCCAo had higher hippocampal levels of p-ERK and lower hippocampal levels of pJNK and pp38. Furthermore, treatments that induced alterations in MAPK signaling resulted in impairments in synaptic plasticity and spatial learning [37, 38]. On the basis of these reports, we conducted the present experiment to determine whether *F. mume *inhibited p-ERK in the hippocampus of rats exposed to chronic BCCAo; we found that the levels of hippocampal p-ERK in the chronic BCCAo hippocampus treated with *F. mume* were decreased, compared with those in the chronic BCCAo hippocampus.

NF-*κ*B plays a critical role in developmental and synaptic plasticity, as well as cell survival [17]. NF-*κ*B essentially consists of two proteins, p50 and p65. In its inactive form, NF-*κ*B is present in the cytoplasm as a homodimer or a heterodimer and is associated with an inhibitory subunit called I*κ*B-*α*. The activation of NF-*κ*B in response to inducers such as proinflammatory stimuli results in degradation of I*κ*B, followed by nuclear translocation of NF-*κ*B. Liu et al. (2006) reported increases of p65 expression in the immunostaining of chronic BCCAo brains, mainly in astrocytes, and low expression in neurons [20]. Moreover, as mentioned previously, *F. mume *inhibited NF-*κ*B activation in LPS-stimulated RAW 264.7 cells. Hence, the present study examined the status of cytosolic I*κ*B-*α*, cytosolic NF-*κ*B p65, and nuclear NF-*κ*B p65 in the chronic BCCAo hippocampus and in the chronic BCCAo hippocampus with treatment of *F. mume*; levels of cytosolic I*κ*B-*α* and cytosolic NF-*κ*B p65 were decreased and that of nuclear NF-*κ*B p65 were increased in the chronic BCCAo hippocampus relative to those in the control hippocampus, indicating increased activity of NF-*κ*B in response to BCCAo. The activity of NF-*κ*B was not increased in the chronic BCCAo hippocampus treated with *F. mume, *compared to those in the chronic BCCAo hippocampus.

Microglial activation was clearly observed in the hippocampus, particularly CA1, and hippocampal fimbria in BCCAo brains, which was reduced in the BCCAo hippocampus by *F. mume *treatment. And the present experiment showed that activation of MAPK signaling and NF-*κ*B translocation induced by chronic BCCAo occurred in hippocampal CA1 and CA3 neurons. However, further studies need to examine whether activation of MAPK signaling and NF-*κ*B translocation occur in microglia or in blood vessels in the hippocampal area with chronic BCCAo, and whether reduction of BCCAo-induced MAPK and NF-*κ*B activity by treatment of anti-inflammatory agent, such as *F. mume, *occur mostly in neurons or the other cells. 

## 5. Conclusion

Microglial cells were activated, and phosphorylation of ERK and NF-*κ*B activity was increased in the chronic BCCAo hippocampus. Treatments with* F. mume* extract reduced the phosphorylation of ERK, NF-*κ*B activity, and microglial activation in the chronic BCCAo hippocampus. These treatments also improved the spatial memory impairments induced by chronic BCCAo. These results suggest that *F. mume* could be a potent new anti-inflammatory treatment for VaD and other CNS diseases.
